# *Globularia alypum* Extracts Attenuate Hyperglycemia and Protect against Various Organ Toxicities in Alloxan-Induced Experimental Diabetic Rats

**DOI:** 10.1155/2022/6816942

**Published:** 2022-08-30

**Authors:** Mohamed Tiss, Khaled Hamden

**Affiliations:** Laboratory of Bioresources Integrative Biology and Exploiting, Higher Institute of Biotechnology of Monastir, University of Monastir, Monastir, Tunisia

## Abstract

In this study, we attempted for the first time to determine the phytochemical compositions and biopharmaceutical properties of *Globularia alypum* methanol extract (GAME) and *Globularia alypum* water extract (GAWE). High-performance liquid chromatography with diode array detection (HPLC-DAD) analysis was performed to establish the chemical profile of the investigated extracts. Chemical composition analysis was taken in the presence of various bioactive compounds such as quercetin 7-O-glucoside and apigenin 7-O-glucoside in GAME. In GAWE, various abundant compounds were found in the extract such as quercetin 7-O-glucoside, apigenin, quercetin, apigenin 7-O-glucoside, and cinnamic acid. This study showed that the administration of GAWE and GAME to type 1 diabetic rats decreased fasting blood glucose, protected pancreas *β*-cells from death and injury, increased liver glycogen rate, and ameliorated oral glucose tolerance test. Moreover, GA reduced weight loss, and diabetes decreased basic physical activity. In addition, the administration of GA extracts in diabetic rats protected from diabetes-induced liver, kidney, testes, heart, and bone toxicities. *Conclusion.* GAWE has possible value for antidiabetic oral medication.

## 1. Introduction

Diabetes caused a serious impact on the lives and well-being of individuals, families, and societies worldwide. It is among the top 10 causes of death in adults and was estimated to have caused four million deaths globally in 2017 [1]. In 2017, global health expenditure on diabetes was estimated to be USD 727 billion [1]. Type 1 diabetes is a metabolic disease characterized by a decline in insulin level and activity and consequently glucose and lipid metabolism syndromes. Actually, more than 700 million (over 6.3%) suffered from type 1 diabetes, and the prevalence increased from 463 million in 2019 to 700 million in 2045 [2]. Regularly increased blood glucose is the cause of various diseases and perturbation such as liver toxicity, neuropathy, perturbation of lipid profile, and inflammation [3–10].

In recent years, it has reported that plant-food polyphenols maybe good supplementary food for treatments of various diseases such as diabetes, obesity, inflammation, and other diseases [3–10]. The beneficial effects of medicinal plants were largely attributed to various compounds such as flavonoids, polyphenols, and phenolic acids [11, 12]. These compounds exert various beneficial actions for human health. Previous studies have reported that the consumption of vegetable and phenolic compounds has been used for the prevention and treatment of various perturbations including hyperglycemia, hyperlipidemia, hypertension, and others perturbations and diseases [13–15].

G. alypum is a medicinal plant that contains many secondary metabolites including flavonoids, tannins, and anthocyanins, which give it several benefits and therapeutic properties [16–18]. Therefore, the goal of the present study is to investigate for the first time the effect of GAME and GAWE on hyperglycemia, pancreas, liver, kidney, testes, and bone tissue toxicities in the alloxan model diabetic rats. In addition, the phytochemical contents of GAME and GAWE were also targeted.

## 2. Materials and Methods

### 2.1. Chemicals and Reagents

Alloxan monohydrate, formic acid, potassium hydroxide, sulfuric acid, ethanol, and acetonitrile were purchased from Sigma Chemical (St. Louis, MO).

### 2.2. Plant Material

The leaves of GA were collected in March 2020 from the region of Sfax in Tunisia and were identified by a specialist of Botany, High School of Biotechnology, Monastir. The leaves were dried at room temperature, powdered, and extracted by maceration in methanol or water (20 g/100 ml) at room temperature for 72 h under mechanical agitation. Thereafter, the extract was filtered with filter paper and concentrated with a rotary evaporator at 45°C to obtain solid residues. The dried GAWE and GAME were kept in the dark at 3°C for further analysis and animal experimentation.

### 2.3. HPLC-DAD Analysis

The detection and the level of phenolic compounds in GAWE and GAME were studied by reversed-phase HPLC analysis using a binary gradient elution according to our previous publication [19].

### 2.4. Induction of Diabetes

Male Wistar rats aged 3 months and weighed 169 ± 9g were used in this experimentation. The rat's experimentation was conducted according to the Guide for the Care and Use of Laboratory Animals (Code: 86/609/EEC). Diabetes was induced by a single alloxan injection at a dose of 135 mg/kg, and the rats were kept for the next 24 h on 20% glucose solution to prevent hypoglycemia. Normal rats were received physiological saline by injection. Seven days later, the rats with blood glucose levels of ≥2 g/l were used for the following study.

### 2.5. Experimental Procedure

A total of 35 rats (28 surviving diabetic rats and 7 control animals) weighed were used for this experimentation and were subdivided into five groups. Group 1: is designated as the normal rats (NC). Group 2 is named the diabetic rats (*D*). Group 3 is named GAME-treated diabetic (*D* + GAME) by gastric gavage method at a dose of 200 mg/Kg bw [20]. Group 4 is named GAWE-treated diabetic rats (*D* + GAWE) by gastric gavage method at a dose of 200 mg/Kg bw [20]. Group 5 is named glibenclamide-treated diabetic rats (*D* + GB) at a dose of 10 mg/kg by gastric gavage method. One month later, all rats were sacrificed and the blood was collected. The pancreas, liver, kidney, testes, and bone were removed and cleaned and stored in formol 10% for histological study.

For the OGGT test, normal, untreated diabetic, and GAME, GAWE, or GB-treated rats received 2 ml glucose solution (2 g/ml/rat) orally via gavage. Blood glucose levels were measured at 0, 30, 60, 90, and 120 min subsequently to receive glucose by glucometer.

### 2.6. Biochemical Analysis

The *α*-amylase activity and serum glucose level were assayed by the determination of the level of glucose obtained from the CNPG3 substrate (Kits Biomaghreb, Tunisia, ref 20033). The lipase activity in the pancreas and the small intestine was measured by the determination of the level of free fatty acids released from triglyceride substrate (Kits Biomaghreb, Tunisia, ref 20132). The serum TC, LDL-C, and HDL-C rates were quantified by using commercial kits from Biomaghreb (Ref 95516).

### 2.7. Statistical Analysis

Tables and figures are presented as means ± standard deviation. The differences between all groups were evaluated using ANOVA and the Fisher test (StatView, version 5), and the significant variation was considered at *p* ≤ 0.05.

## 3. Results

### 3.1. Characterization of Phytochemicals by HPLC-DAD Analysis

**Figure 1:**
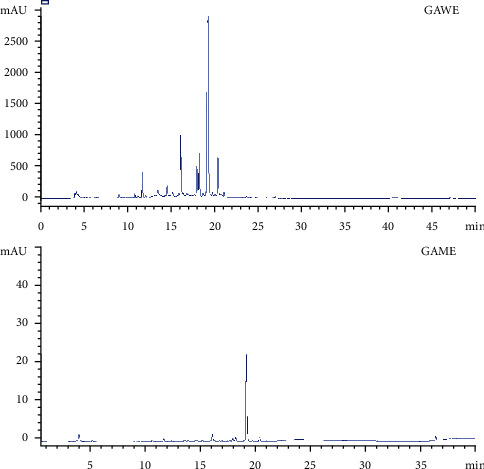
HPLC chromatogram of water (a) and methanol (b) extracts from leaves of *GA*.

Eleven compounds were identified or tentatively characterized in the extracts of G. alypum leaves. HPLC-DAD analysis showed the presence of three major phenolic compounds in GAME: quercetin 7-O-glucoside, vanillic acid, and apigenin. In GAWE, our study showed the presence of seven major phenolic compounds, namely, quercetin 7-O-glucoside, apigenin 7-O-glucoside, apigenin, quercetin, cinnamic acid, protocatechuic acid, and p-coumaric acid. These compounds were identified by their retention times and UV spectra (Figure 1, Table 1).

### 3.2. Acute Toxicity Studies of GAME and GAWE

The GAME and GAWE did not provoke any toxicity up to a dose of 2 g/kg. GAME and GAWE did not provoke a significant change in animal comportment or mortality until the dose of 400 mg/kg.

### 3.3. Effect of GAME or GAWE Supplementation on Basic Physical Activity

This study showed that diabetes or hyperglycemia provoked a lowering effect of physical activity as compared to normal rats. Also, the supplementation of GAME or GAWE to diabetic rats restores physical activity. The positive effects of GAWE and GAME were observed until 140 min after administration.

### 3.4. Effect of GAME and GAWE Extracts on the Pancreas of Diabetic Rats

**Figure 2:**
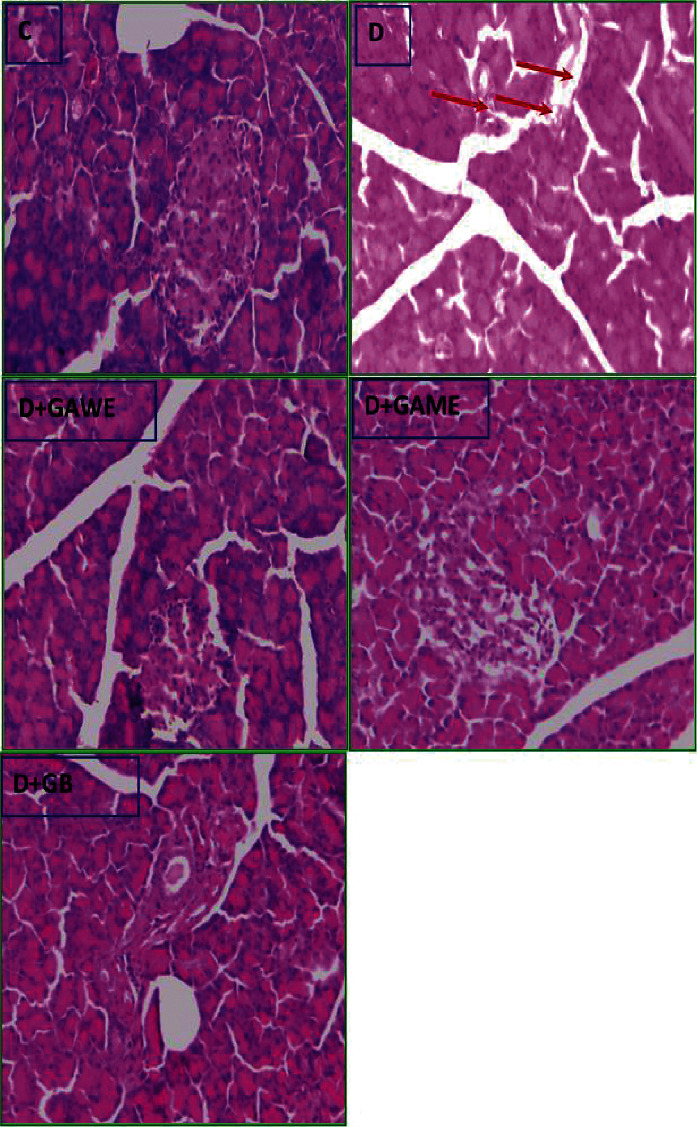
Effect of GAWE and GAME on blood glucose level, liver and muscle glycogen levels, and OGGT. The administration of GAWE or GAME to diabetic rats decreases serum glucose rate, increases the muscle and liver glycogen levels, and ameliorates the insulin sensitivity. The values are statistically presented as follows: ^@^*p* < 0.05 as compared to normal rats; ^#^*p* < 0.05 as compared to alloxan-induced type 1 diabetes; ^&^*p* < 0.05 as compared to GAWE diabetic treated rats.

The histological examination of pancreas tissues showed normal islets in the control rats (C); however, the pancreas of diabetic rats noted clear damage and death of pancreas *β*-Cells (*D*). In addition, the supplementation of GAME and GAWE to diabetic rats protects *β*-Cells from death. Conversely, a clear regenerative action of *β*-cells was shown after the supplementation of GAWE or GAME (Figure 2).

### 3.5. Effect of GAME and GAWE Extracts on Body Weight and Food and Water Consumption

In the normal rats, there is an increase in body weight; however, food and water consumption was unchanged during three weeks of treatment. In untreated diabetic rats, a decrease in body weight and an increase in water and food consumption were shown. In GAME- and GAWE-treated diabetic rats, a slight decrease in body weight was observed, whereas food and water consumption markedly increased as compared to normal rats (Table 2).

### 3.6. Effect of GAME and GAWE Extracts on Serum Blood Level and Liver Glycogen Rate

**Figure 3:**
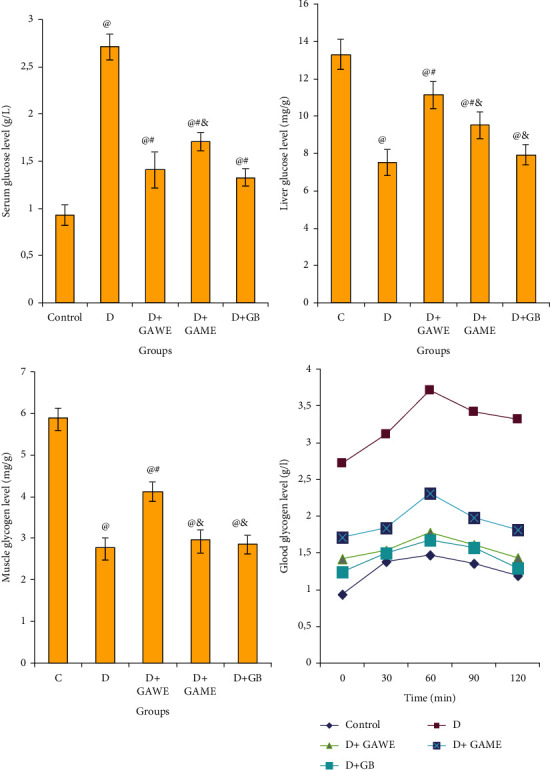
Effect of GAWE and GAME on alloxan-induced *β*-cells death staining by H&E (100 (X). C: normal *β*-cells; D: alloxan-induced severe *β*-cells death; (*D* + GAWE and *D* + GAME): a protective effect was shown in the pancreas of diabetic rats treated with GAWE or GAME. The protective effect was more pronounced in GAWE diabetic treated rats.

Figure 3 evaluates the action of GAME and GAWE supplementation to alloxan-induced type 1 diabetes rats on blood glucose level and liver glycogen content. In fact, the supplementation of GAWE and GAME to alloxan-induced diabetes in rats decreased serum glucose rate by 47 and 36% as compared to the control diabetic rats, respectively (Figure 3). In addition, a significant reduction in the diabetic rats' liver and muscle glycogen levels was shown. However, the supplementation of GAWE and GAME to the diabetic rats by gastric gavage route reestablished the liver and muscle glycogen rate (Figure 3).

### 3.7. Effect of GAME and GAWE Extracts on OGTT

In normal rats, this study showed that the serum glucose rate was augmented to a maximum of 1.47 g/L after 1 hour of oral glucose supplementation and to feedback to standard level 120 min later (Figure 3). In diabetic rats, the supplementation of 2 g/mL glucose increased the blood glucose level, with a peak of 3.71 g/L 60 min later. In treated diabetic rats, the supplementation of GAWE and GAME by gastric gavage route reduced the peak glucose rates from 3.71 to 1.77 and 2.31 g/L, respectively (Figure 3).

### 3.8. Effect of GAME and GAWE Extracts on Diabetes-Induced Liver Tissue Toxicity

**Figure 4:**
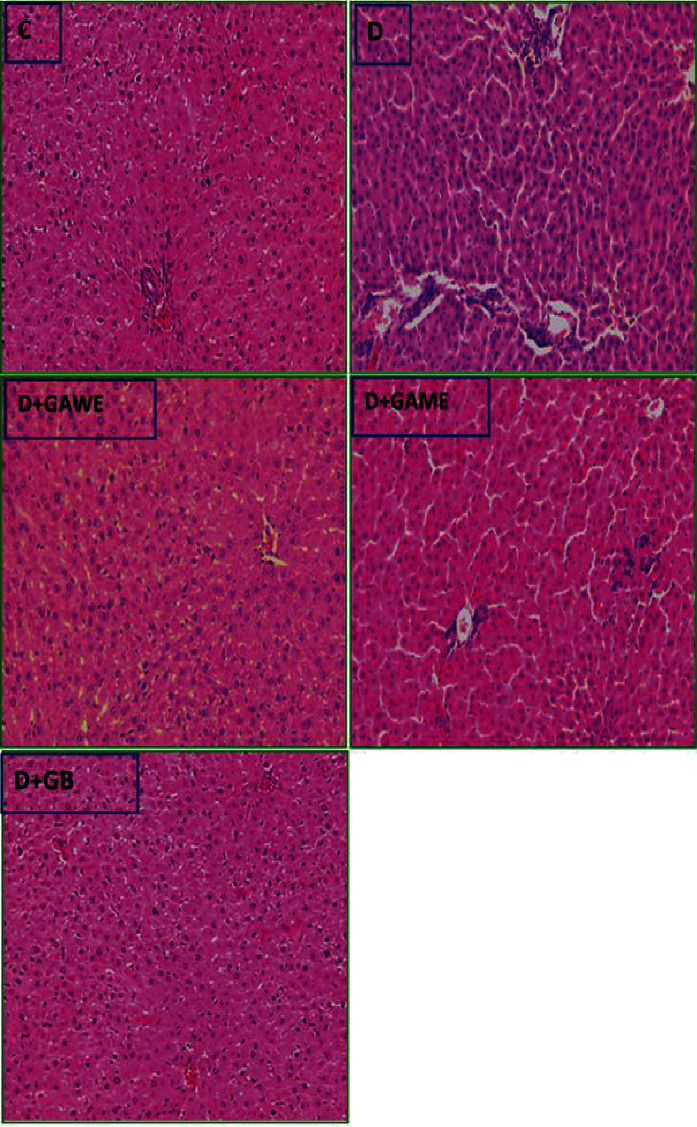
Effect of GAWE and GAME supplements on diabetes-induced liver injury. C: normal liver tissues and architecture. D: the liver of alloxan-induced type 1 diabetes-hyperglycemia-induced apparition lymphocytic infiltrate in the portal and sinusoidal spaces. D + GAWE and D + GAME: diabetic rats treated with GAWE or GAME; prevention of liver-infiltrating lymphocytes (H&E 100X).

The table demonstrates that diabetes induced a mild to moderate portal inflammation minimal hepatocyte necrosis and the apparition lymphocytic infiltrate in the sinusoidal infiltration (*D*). However, the supplementation of GAME and GAWE to alloxan-induced type 1 diabetes in rats defends liver tissue inflammation and toxicity evidenced by the reduced lymphocytes infiltration phenomena (Figure 4).

### 3.9. Effect of GAME and GAWE Extracts on Diabetes-Induced Kidney Lymphocytes Infiltration

**Figure 5:**
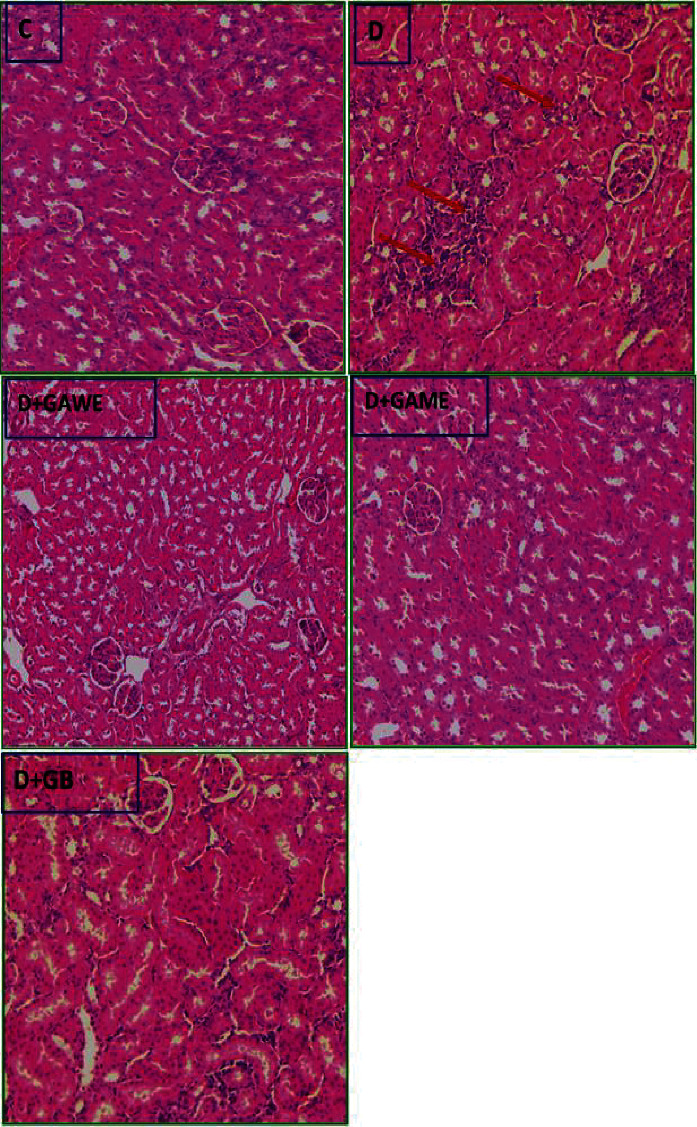
C: normal rat kidney. D: diabetes-provoked inflammation in kidney tissues. D + GAWE and D + GAME: administration of GAWE or GAME to alloxan-induced type 1 diabetes reverted and inhibited inflammation in kidney tissues (H&E 100X).

The table demonstrates that hyperglycemia induced kidney tissue alteration evidenced by alteration collecting system and interstitial lymphocytic infiltration. However, the treatment of diabetic rats by GAME or GAWE protects them from diabetes-induced kidney toxicities showed by the absence of kidney-infiltrating lymphocytes and the alteration of the renal collecting system (Figure 5).

### 3.10. Histological Alteration in Testes Diabetic Rats Treated with GAME or GAWE

**Figure 6:**
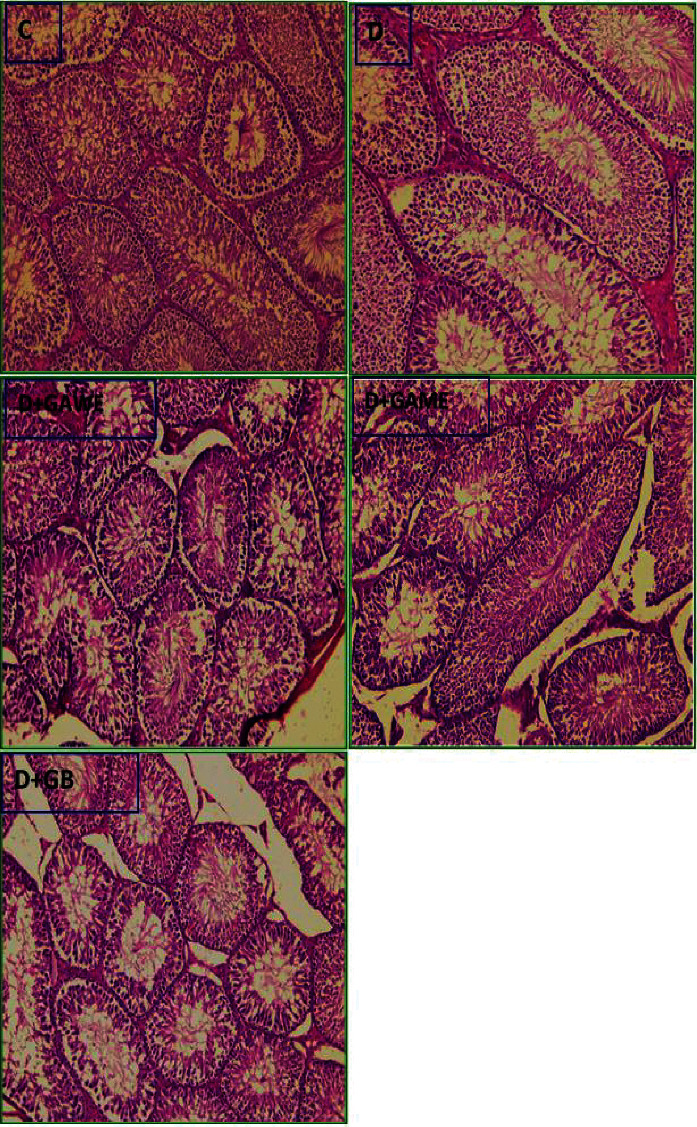
Effect of type 1 diabetes on the histological morphology of rat testes (400 (X). C: control rats showing a regular development of spermatogenesis. D: diabetic rats showing decrease in sperm count in somniferous tubules. In GAME- and GAWE-treated rats, spermatogenesis proceeded normally and somniferous tubules sperm count was near to that of normal rat germ cells.

In diabetic rats, a weakening of germ cells and spermatozoids in somniferous tubules was observed. In diabetic rats treated with GAME or GAWE, spermatogenesis proceeded normally and somniferous tubules sperm count was similar to that of normal rats (Figure 6).

### 3.11. Histological Changes in Heart Diabetic Rats Treated with GAME and GAWE

**Figure 7:**
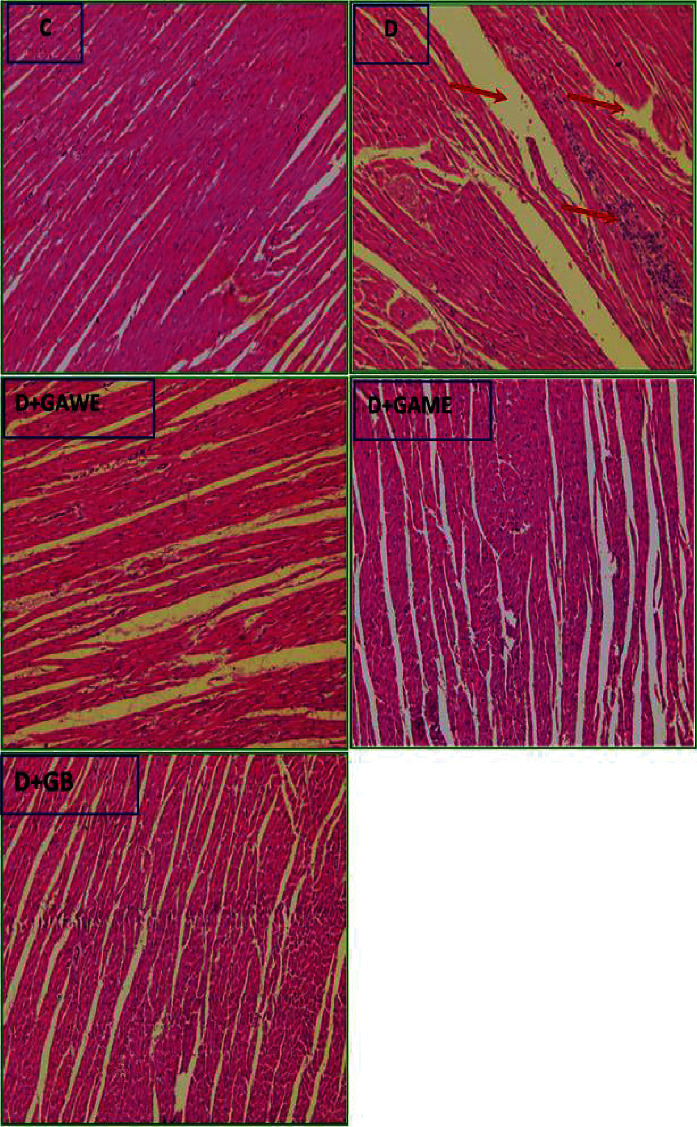
Effect of GAWE or GAME on type 1 diabetes cardiac injury in rats. (C) Control groups: normal cardiac architecture. D: alloxan-treated group showing anarchized myocardial fibers. D+GAWE and D+GAME: diabetic group received GAWE or GAME (200 mg/kg) showing prevention and amelioration of anarchized myocardial fibers.

Microscopic analysis of normal rats showed regular cardiac architecture of tissues and muscles with regularly arranged cardiac myofibers. Histological analysis of heart tissues of diabetic rats showed alteration of heart tissues observed by disorganized myocardial fibers and lymphocyte infiltrate. In the heart of GAWE diabetic treated rats, the lymphocyte infiltration has disappeared but disorganized myocardial was persisted. In the heart of GAME diabetic treated rats, the myocardial fiber arrangement similar to that of the normal rats and the lymphocyte infiltration were disappeared (Figure 7).

### 3.12. Effect of GAME and GAWE Extracts on Diabetes-Induced Bone Toxicity

**Figure 8:**
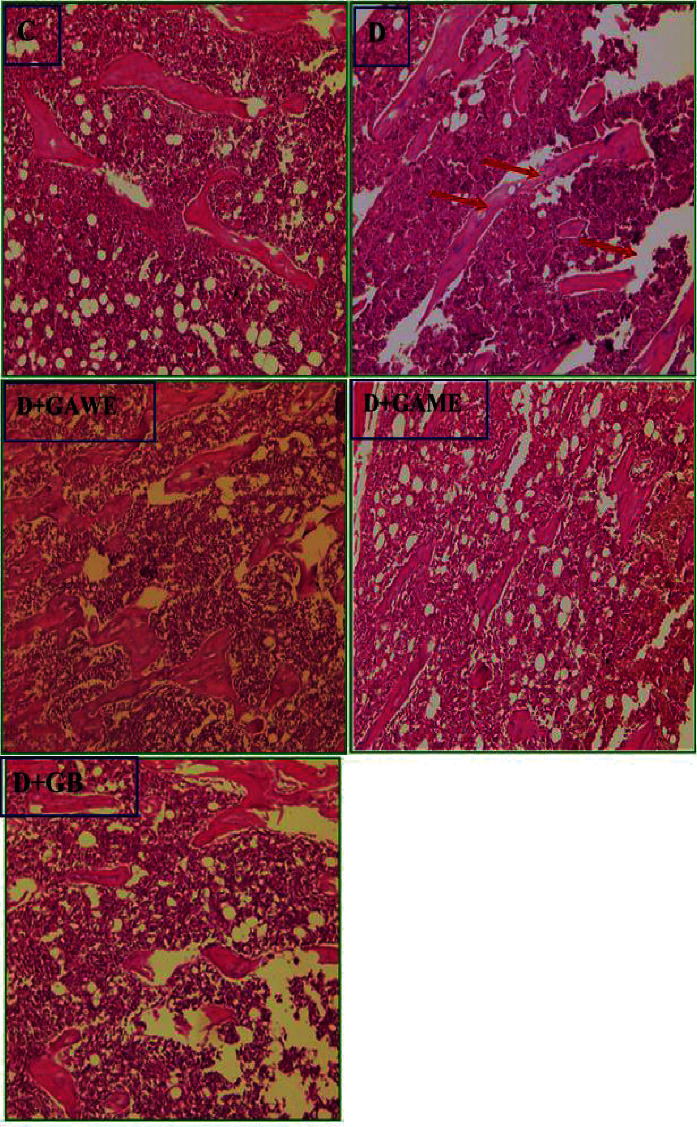
C: bone and normocellular bone marrow. D: in diabetic rats, we showed a spongy bone is composed of a network of trabeculae separated by interconnecting spaces. In diabetic rats treated with GAME or GAWE, the bone tissues were similar to that of the control group.

Figure 8 demonstrates a normal bone tissue architecture in normal rats. In diabetic rats, we showed a spongy bone is composed of a network of trabeculae separated by interconnecting spaces containing normocellular bone marrow. The supplementation of GAME or GAWE to diabetic rats protects from diabetes-induced bone alteration and the bone tissues similar to that of the control group (Figure 8).

## 4. Discussion

The HPLC-DAD analysis showed the existence of various phenolic acids and flavonoids in GA extracts. GAME and GAWE contain high quantities of quercetin 7-O-glucoside and apigenin 7-O-glucoside. The highest amounts of quercetin 7-O-glucoside and vanillic acid were revealed in GAME, while gentisic acid, catechin, 4-hydroxybenzoic acid, protocatechuic acid, cinnamic acid, p-coumaric acid, apigenin 7-O-glucoside, apigenin, and quercetin were higher in GAWE than that in GAME.

In the present study, the antihyperglycemic and the protective effect of various perturbations provoked by diabetes were evaluated. *G. alypum* methanol extract (GAME) and G. alypum water extract (GAWE) were studied against alloxan-induced type 1 diabetes mellitus in rats. Diabetes in the alloxan-induced hyperglycemic rats was confirmed here by blood glucose level, body weight loss, OGTT, and physical performance on the 7th-day postalloxan injection.

In this study, the injection of alloxan to rats potentially induced *β*-cells damage and death (Figure 3); consequently, increase in blood glucose rate and reduced glycogen level in the liver and muscle were observed. The supplementation of GAWE or GAME protected pancreas *β*-cell from damage and death. This protection of pancreas *β*-cells from death after the supplementation of GAME or GAWE to surviving diabetic rats may be mediated by the stimulation of *β*-cell regeneration. In fact, Figure 3 showed that pancreas tissue was completely destroyed in alloxan-treated rats, and this destruction was minimized in GAWE and GAME diabetic-treated rats. According to the previous research, GA stimulated the insulin secretion in islets of Langerhans and inhibited starch digestive enzymes such as *α*-amylase and *α*-glucosidases [21, 22]. The powerful protective effect of pancreas *β*-cells may be influenced by the presence of a variety of bioactive compounds in GAWE such as quercetin 7-O-glucoside, apigenin 7-O-glucoside, apigenin, quercetin, cinnamic acid, protocatechuic acid, and p-coumaric acid (Table 1). In GAME, HPLC-DAD analysis showed the presence of three major phenolic compounds in GAME, namely: quercetin 7-O-glucoside, vanillic acid, and apigenin. Singh et al. [23] have shown that the oral administration of vanillic acid to diabetic rats significantly reduced the hyperglycemia, increased liver enzymes, and normalized lipid profile that was altered in diabetic rats. Moreover, vanillic acid attenuated the impaired renal function as evidenced by a reduction in serum creatinine, urea, uric acid, and urinary microproteinuria levels with a concomitant increase in urinary creatinine clearance in the nephropathic rats [23]. In addition, apigenin administration to diabetic rats decreased the blood glucose level significantly [24].

GAWE was shown to be rich in phenolic content such as quercetin, quercetin 7-O-glucoside, vanillic acid, and apigenin, which are largely responsible for their numerous medicinal properties including blood glucose-lowering effect. Research by Imessaoudene et al. [25] has shown that quercetin-reduced blood glucose level in diabetes is attained through the inhibition of glucose absorption in the intestine and increased absorption by peripheral tissues. Quercetin also accelerates the use of glucose in the liver and skeletal muscle cells by activating key glycolysis enzymes, hexokinase and pyruvate kinase, reducing the activity of glycogen phosphorylase, and stimulating glycogen synthesis in the liver and skeletal muscle [26]. Ravikumar and Kavitha [27] have reported that quercetin treatment showed a dose-dependent decrease in alloxan-induced hyperglycemia with a significant reduction at 30 mg/kg. Kumari et al. [28] demonstrated that the treatment of diabetic rats with vanillic acid markedly attenuated STZ-induced body weight loss and hyperglycemia, along with improved lipid profile and HbA1c, without significant alteration of serum insulin levels.

Liver and muscles are major tissues for glucose utilization in the mediation of insulin. In this study, the administration of alloxan to rats induced pancreas *β*-cells death (Figure 3); and therefore, a decline in insulin level and secretion. This caused a lack of glucose uptake in the liver and the muscles, leading to decreases in the glycogen level (Figure 2) and increases in blood glucose levels (Figure 2). The administration of GAME or GAWE to diabetic rats, however, protects pancreas *β*-cells from death and damage; and this increases insulin secretion and regained glycemic control, augmented glycogen level in the liver and muscle, and ameliorated oral glucose tolerance test (Figure 2).

Interestingly, this study showed that hyperglycemia decreased physical activity in alloxan-induced type 1 diabetes. In addition, we noted that the supplementation of GAME or GAWE to diabetic rats stimulates physical activity. In addition, type 1 diabetes is associated with alterations in the general body such as weight loss and an increase in water and food consumption. In fact, previous studies have reported insulin deficiency-induced catabolism of proteins and muscle wasting and consequently loss *n* body weight and increased diet and water consumption [29, 30]. The administration of GAME or GAWE to diabetic rats improved insulin level and activity and reduced these general alterations.

This study demonstrated that rats with type 1 diabetes presented liver and kidney tissue lesions and alterations showed by the apparition lymphocytic infiltrate in the liver and alteration collecting system and interstitial lymphocytic infiltration in the kidney tissues. This study also showed that the supplementation of GAWE or GAME to diabetic rats decreased serum glucose levels. The therapeutic effect of the two extracts may also be due to the GAWE or GAME active flavonoids, phenols, steroids, and saponins. In fact, these polyphenols may protect *β*-cells from death, stimulate regeneration of pancreas cells, and stimulate insulin secretion and activity. GAWE extract containing various phenolic compounds such as quercetin 7-O-glucoside, apigenin 7-O-glucoside, and vanillic acid regenerates pancreas *β*-cell, and therefore increased insulin secretion, and prevented hyperglycemia [31–33].

Previous studies have reported that type 1 diabetes declines fertility rate [34, 35]. Results of this study showed a scarcity of spermatogenesis with decreased sperm count when compared to control rats. The administration of GAWE and GAME protects against testes damage and decreases spermatozoid count in somniferous tubules. These results suggest that an increase in blood glucose level was associated with a significant decrease in spermatozoid count; and the administration of GAWE and GAME significantly decreased blood glucose level, and this could significantly leading to increase in sperm count in the testis.

Results of this study showed that diabetes-induced myocardial fiber disorganization and a spongy bone are composed of a network of trabeculae separated by interconnecting spaces containing normocellular bone marrow. However, administration of GAME and GAWE is highly effective in preventing tissue damage by reducing alteration of heart and bone tissues. These results are consistent with previously reported studies that supplementation of herbal flavonoids and polyphenols to diabetes-induced heart damage improved and minimized cardiac injury [36, 37].

## 5. Conclusion

Results of the current study demonstrates, for the first time, that water extract of this plant exhibited promising beneficial effects for the prevention and improvement of diabetes and protected from hyperglycemia-induced various organ toxicities. They also suggest that GA can be safely and fruitfully used in future therapeutic and medicinal applications as a therapeutic agent for the treatment of diabetes, liver, and kidney dysfunctions and fertility. Accordingly, further studies are currently under way in our laboratories to further explore this antidiabetic agent and to make its application suitable for the therapeutic and medicinal industries interested in the development of antidiabetic natural drugs.

## Figures and Tables

**Table 1:** Phenolic compounds identified by HPLC-DAD analysis in methanolic extract (GAME) and aqueous extract (GAWE) of *G. alypum* leaves.

Peak compound	RT (mn)	Compound	GAWE (%)	GAME (%)
1	4.42	Gentisic acid	2.4	2.1
2	5.23	Catechin	2.56	1.09
3	6.82	4-Hydroxybenzoic acid	1.4	1.39
4	6.98	Protocatechuic acid	3.95	1.37
5	8.51	Vanillic acid	2.52	3.9
6	11.79	Cinnamic acid	8.31	1.11
7	14.27	p-Coumaric acid	3.35	1.87
8	16.12	Apigenin 7-O-glucoside	13.51	12.3
9	18.82	Apigenin	11.31	2.71
10	19.15	Quercetin 7-O-glucoside	35.71	45.51
11	20.38	Quercetin	10.77	1.07

**Table 2:** Average body weight (g), food intake (g/rat), and water in GAWE and GAME type 1 diabetes treated rats. In diabetic rats, a decrease in body weight and an increase in water and food consumption were observed.

	Body weight			Food consumption (g/rat/day)			Water consumption (ml/rat/day)		
	W1	W2	W3	W1	W2	W3	W1	W2	W3
Nondiabetic (NC)	181 ± 11	197 ± 13	221 ± 13	19.71 ± 3	18.73 ± 5	20.13 ± 5	18.7	19.3	19.73
Diabetic (D)	169 ± 9	157 ± 10	143 ± 11	26 ± 5	31 ± 11	35 ± 5	41 ± 3	47 ± 2	48 ± 7
D + GAWE	175 ± 7	183 ± 9	209 ± 8	23.1 ± 3	22.1 ± 4	20.7 ± 7	25 ± 5	24 ± 3	23 ± 3
D + GAME	171 ± 9	180.7 ± 7	197.1 ± 6	27.1 ± 7	25.9 ± 3	24.6 ± 4	29.1 ± 7	27.3 ± 6	25 ± 4
D + GB	175 ± 7	181 ± 3	203 ± 5	25.1 ± 5	24.1 ± 7	22.3 ± 5	27 ± 3	25 ± 4	24 ± 2

In GAME- or GAWE-treated diabetic rats, a minor decrease in body weight was observed; however, food and water consumption increased.

## Data Availability

No data were used to support this study.
